# The interplay between Helicobacter pylori infection and rs738409 PNPLA3 in metabolic dysfunction-associated steatotic liver disease

**DOI:** 10.1371/journal.pone.0310361

**Published:** 2024-09-23

**Authors:** Facundo Maiorana, Magali Neschuk, María Virginia Caronia, Karina Elizondo, María Laura Robledo, Adolfo Schneider, Georgina Veron, Pedro Dario Zapata, Fernando Javier Barreyro

**Affiliations:** 1 Laboratorio de Biotecnología Molecular (BIOTECMOL), Instituto de Biotecnología de Misiones “Dra. María Ebbe Reca” (InBioMis), Facultad de Ciencias Exactas Químicas y Naturales, Universidad Nacional de Misiones, Misiones, Argentina; 2 Fundación HA Barceló, Instituto Universitario en Ciencias de la Salud, Santo Tomé, Corrientes, Argentina; 3 Área de Biología Molecular, Servicio de Patología, Hospital de Pediatría “Prof. Dr. Juan P Garrahan”, Ciudad Autónoma de Buenos Aires, Buenos Aires, Argentina; 4 CONICET, Buenos Aires, Argentina; Tehran University of Medical Sciences, ISLAMIC REPUBLIC OF IRAN

## Abstract

**Background:**

Recent studies have suggested an association between *H*. *pylori* and metabolic-disfunction associated fatty liver disease (MASLD). However, epidemiologic studies have yielded inconsistent results. We aim to evaluate the association of *H*. *pylori* and G-allele PNPLA3 in MASLD diagnosis, and markers of severity.

**Methods:**

A multi-center cross-sectional study was conducted. A total 224 functional dyspepsia (FD) patients cohort who underwent gastroscopy was selected. Biochemical, clinical parameters, ultrasound, FIB-4 score, LSM by VCTE, gastric biopsies, *H*. *pylori* status, and rs738409 PNPLA3 were evaluated. A second retrospective cohort of 86 patients with biopsy-proven MASLD who underwent gastroscopy with gastric biopsies was analyzed.

**Results:**

In the FD cohort MASLD was observed in 52%, and *H*. *pylori*-positive in 51%. *H*. *pylori* infection was associated with MASLD prevalence, but in multivariate analyses adjusted for G-allele PNPLA3, it became not significant. Then in MASLD-only dyspeptic cohort, *H*. *pylori* infection was significantly linked to elevated serum AST levels and increased liver stiffness measurements, suggesting a potential role in liver injury and fibrosis. Histopathological analysis in biopsy-proven MASLD patients further supported these findings, showing a significant association between *H*. *pylori* infection and increased NAS score, fibrosis stage, and prevalence of MASH. Notably, the combination of *H*. *pylori* infection and G-allele PNPLA3 appeared to exacerbate MASLD severity beyond individual effects.

**Conclusions:**

Our results suggest that *H*. *pylori* infection may play a role in the progression of liver injury and fibrosis in patients with MASLD, especially in those with specific genetic predispositions.

## Introduction

Metabolic-dysfunction-associated steatotic liver disease (MASLD), previously known as non-alcoholic fatty liver disease (NAFLD), has emerged as a significant public health concern globally and is currently the most common chronic liver disease [1]. The global prevalence of MASLD has been steadily increasing in parallel with the rising rates of obesity and metabolic comorbid disease such as metabolic syndrome and type-2 diabetes mellitus [2, 3]. MASLD encompasses a spectrum of liver disorders ranging from simple steatosis to metabolic-dysfunction associated steatohepatitis (MASH), fibrosis, cirrhosis, and hepatocellular carcinoma (HCC) [1, 4, 5]. MASLD is a complex disorder influenced by multiple mechanisms, including genetic, environmental, and metabolic factors [6, 7]. Among the genetic factors implicated in the pathogenesis of MASLD, the single nucleotide polymorphism (SNP) rs738409 in the PNPLA3 gene has attracted considerable attention. Numerous studies have demonstrated a strong association between individuals carrying the rs738409 G-allele in the PNPLA3 and the development and progression of MASLD [8–10]. This SNP results in the substitution of isoleucine for methionine at position 148 (I148M) in the PNPLA3 protein, leading to altered lipid droplet remodeling and increased hepatic triglyceride accumulation [11]. Yet, how this variant predisposes to inflammation and fibrosis is currently unclear [12]. In addition to traditional risk factors such as obesity and insulin resistance, emerging evidence suggests a potential role for microbial dysbiosis and chronic low-grade inflammation in the development and progression of MASLD [13, 14].

*Helicobacter pylori* (*H*. *pylori*) is a gram-negative, microaerophilic bacteria that colonizes gastric mucosal epithelium and is a major cause of chronic gastritis, peptic ulcers, gastric mucosa-associated lymphoid tissue lymphoma, and non-cardia gastric adenocarcinoma [15, 16]. *H*. *pylori* is also the main infectious-related ethological cause of functional dyspepsia (FD) [17]. Indeed, dyspeptic symptoms associated with *H*. *pylori*, as per the Kyoto consensus, may also be considered as a separate entity [18]. Similarly to the high prevalence of MASLD, *H*. *pylori* has highly global prevalence, affecting nearly 50% of the world’s population, with a higher prevalence in developing countries, and is a key constituent of the human microbiome [15, 19, 20]. *H*. *pylori* infection not only affects gastric mucosa but is also linked to a number of extra-gastric diseases, indicating that *H*. *pylori* may cause disease far from the primary site of infection by a different pathogenic process [21]. In recent years, there has been growing interest in the potential association between *H*. *pylori* infection and MASLD, fueled by the overlapping risk factors and shared pathophysiological mechanisms between these two conditions [22–25]. However, the existing literature on the association between *H*. *pylori* infection and MASLD is conflicting, with some studies reporting a positive association while others finding no significant relationship [26–29]. Several studies have suggested that *H*. *pylori* infection may contribute to the pathogenesis of MASLD through various mechanisms, including chronic low-grade inflammation, alterations in gut microbiota composition, and modulation of insulin sensitivity [19, 30–34]. It is noteworthy that *H*. *pylori* infection per se is linked to gut dysbiosis, including alterations in bacterial diversity and abundance that may influence metabolic derangements [32, 35]. Notably, *H*. *pylori* promotes hepatocyte cytotoxicity through internalization of the virulence genes *cagA* and *vacA* [36, 37]. The interaction between *H*. *pylori* and human genetic polymorphisms in the pro- and anti-inflammatory cascade appear to play a role in the host’s susceptibility to *H*. *pylori* gastritis and duodenitis [38–40]. Considering aforementioned data, we can consider that *H*. *pylori* infection may exacerbate the effects of the PNPLA3 rs738409 variant on hepatic milieu, thereby increasing the risk and severity of MASLD. Thus, our aim was to evaluate the association of *H*. *pylori* and rs738409 G-allele in the PNPLA3 in MASLD severity.

## Materials and methods

We consecutively recruited patients newly diagnosed with FD symptoms based on Rome-IV [41] criteria who underwent upper endoscopy at IOT Medical Center (Posadas, Province of Misiones) and University Hospital San Juan Bautista (Santo Tomé, Province of Corrientes), Argentina, were evaluated in this study. We consecutively recruited patients diagnosed with FD who were scheduled to undergo upper gastrointestinal endoscopy from December 14^th^ 2021 to December 15^th^ 2023.

The criteria for inclusion were: (1) age between 18 and 70 years, (2) symptoms meeting Rome-IV criteria. The criteria for exclusion before gastroscopy were: (1) progressive, severe diseases requiring active medical management (e.g. uncontrolled diabetes, congestive heart failure, end-stage renal failure, neurological disease, advanced cancer, or psychiatric disorder), (2) those with known causes of chronic liver diseases and significant alcohol consumption (defined as  ≥ 140 g/week for women and  ≥ 210 g/week for men), (3) autoimmune medical conditions (inflammatory bowel disease, celiac disease, vasculitis, connective tissue disease), (4) patients who had taken steatogenic medications (corticosteroids, tamoxifen, amiodarone, methotrexate, amiodarone), (5) patients who had taken antibiotic within the past 3 weeks and (7) history gastric or bariatric surgery. Patients receiving proton pump inhibitors (PPI), H2-blockers or non-steroidal anti-inflammatory drugs (NSAID) were advised to suspend them 14 days before endoscopy. The criteria for exclusion after upper endoscopy were: (1) evidence of active peptic ulcer disease, (2) evidence of malignant gastric disease, (3) signs of celiac disease, and (4) not available gastric biopsies. The study was approved by local ethical committee (Comité de Ética del Instituto Universitario en Ciencias de la Salud, CEI IUCS Resol N° 252/21). The study was performed in accordance with the ethical standards as laid down in the 1975 Declaration of Helsinki and its later amendments. Written informed consent was obtained from all participants.

### Clinical variables

Clinical conditions and anthropometric variables were obtained by a pre-endoscopy interview and medical records, data regarding age, sex, body mass index (BMI), medication history, including presence of hypertension, type-2 diabetes (type-2 DBT), surgery, malignancy, regular physical activity (≥150min/week of moderate intensity aerobic physical activities in ≥ 3 sessions) and chronic liver disease were collected. Medication history was evaluated by pre-endoscopy interview, medical records and self-reported questionnaire that involved the regular utilization of NSAIDs, PPI, H-2 blockers, anti-hypertensive medications, anti-diabetic medications, alcohol consumption, smoking habits and daily coffee intake. The laboratory parameters were assessed before upper endoscopy: total cholesterol, HDL-cholesterol, LDL-cholesterol, triglycerides, glucose, insulin, homeostasis model assessment-insulin resistance index (HOMA-IR), aspartate aminotransferase (AST), alanine aminotransferase (ALT), gamma-glutamyl transferase (GGT), alkaline phosphatase (ALP), platelets, serum ferritin and viral hepatitis serology (HBsAg, Anti-HBc and Anti-HCV). The FIB-4 index was calculated using the formula: FIB-4 = Age (years)×AST (IU/L)/[PLT(10^9^/L)× √ALT (IU/L)] [42]. Previously published FIB-4 cut-off value were used to rule-out (<1.3) advanced fibrosis (AF) [43]. Cut off of FIB-4 ≥1.3 is recommended to identify patients with MASLD as intermediate-high risk of AF requiring referral to the specialist liver clinic [43].

### Abdominal symptom evaluation

The Izumo Scale is a self-administered questionnaire designed to assess the effects of abdominal symptoms on quality of life over the past week [44]. The survey has been shown to have good internal consistency, reproducibility, as well as good correlations with the visual analogue scale of abdominal symptoms [45], and is routinely used by our group in clinical practice to evaluate abdominal symptoms [40]. All participants completed the EPS and PDS domain of the Izumo questionnaire scale of abdominal symptom-related quality of life at entry [44]. Each question is rated on a 6-point Likert scale from 0 to 5, with higher values indicating more severe symptoms [46]. Domain-specific scores range from 0 to 15 [44]. Higher scores indicate worse symptoms. Since FD comprises EPS and PDS, we evaluated the severity of FD symptoms by adding both domains in a score ranging from 0 to 30 [47]. FD patients satisfied the Rome-IV criteria for the past three months with symptom onset at least six months before diagnosis [41]. FD was divided into two subtypes depending on the symptoms: Epigastric pain syndrome (EPS) is associated with epigastric pain or epigastric soreness that does not necessarily occur after meal ingestion at least one day a week, and postprandial distress syndrome (PDS) is associated with meal-induced early satiety or postprandial fullness at least three days a week.

### MASLD definition

MASLD was defined according to the multi-society expert group consensus statement on new fatty liver disease nomenclature [48], the diagnosis of MASLD required the following: (1) hepatic steatosis detected by ultrasonography; (2) no significant alcohol consumption (defined as <140 g/week for women and <210 g/d for men); (3) the presence of one cardiometabolic risk factor; and (4) no other discernible cause of steatosis.

### Vibration controlled transient elastography

Vibration controlled transient elastography (VCTE) was performed by FibroScan-402 (Echosens, Paris, France) medical device using the M or XL probe as appropriate. Liver stiffness measurement (LSM) by VCTE was assessed after a diagnosis of MASLD by ultrasound, by an expert operator. Measurements were performed on the right lobe of the liver through intercostal spaces guided by ultrasonography with the patient lying in dorsal decubitus with the right arm in abduction. LSM was expressed in kilopascal (kPa) and calculated as the median value of ten successful acquisitions, defined by a success rate of at least 60%, and by an interquartile range lower than 30% [49]. Previously published LSM cut-offs were used to rule-out Fibrosis stage-2 or greater stage (significant fibrosis, SF) ≥5.8 kPa, and to rule-out Fibrosis stage-3 or greater stage (advanced fibrosis, AF) ≥8 kPa respectively [50–52].

### Endoscopy

All recruited participants underwent upper gastrointestinal endoscopy performed by experienced endoscopists (FJB and AS). Upper endoscopy was done using Pentax EG-2990i series scope. Biopsy specimens were collected from the lesser curvature of the gastric body (two biopsies) and lesser curvature of the gastric antrum (two biopsies) using a Radial Jaw 3 forceps (Boston Scientific, MA, USA).

### Histopathologic analysis

Gastric biopsies were fixed in 10% formalin and processed to paraffin embedding for hematoxylin and eosin (HE) staining by routine methods. The presence of *H*. *pylori* was assessed on gastric biopsies using Giemsa staining in all patients.

### Biopsy-proven MASLD population

Our cohort comprised 101 patients who underwent liver biopsy by medical indication for MASLD evaluation between January 2018 and December 2023 at the Liver Unit, IOT Medical Center (Posadas, Province of Misiones). Grading and staging of MASLD were determined using the NASH Clinical Research Network scoring system [53]. MASH was defined as NAS (NAFLD Activity Score) ≥ 4. Fibrosis staging was performed according to the Kleiner classification [54]. Significant fibrosis was defined as Fibrosis stage ≥2. All patients underwent evaluation for *H*. *pylori* infection via upper endoscopy and gastric biopsies performed within a range of 6 months after the liver biopsy.

### Extraction and Polymerase Chain Reaction (PCR)

DNA extraction from gastric biopsies was carried out following the manufacturer’s instructions with the ADN PuriPrep-T kit (InbioHighway, Argentina). The extracted DNA was stored at −20°C until used. Polymerase Chain Reaction (PCR) was then performed by using specific primers. Target gene, amplicon size, primer names, and sequences are presented in Table 1. For the PCR amplification, 50 ng of DNA was added to a PCR mixture containing 20 μmol forward and reverse primers, 15 μL of MINT Master Mix2x (InbioHighway, Argentina) to the total volume of 25 μL. The PCR conditions, optimized for each primer set (as outlined in Table 1), included an initial denaturation at 94°C for 4 minutes, followed by 35 cycles of denaturation at 95°C for 30 seconds, annealing for 30 seconds (specific temperatures listed in Table 1), an extension at 72°C for 30 seconds, and a final extension at 72°C for 5 minutes, using a Labnet MultiGene MiniThermocycler. The PCR products were then subjected to electrophoresis on a 2% agarose gel (InbioHighway, Argentina) and the resulting bands were visualized using Eco-Gel staining (InbioHighway, Argentina). To define the rs738409 *PNPLA3* genotype, we performed a restriction fragment length PCR [55]. NlaIII restriction enzyme (ThermoFisher Scientific, Waltham, MA, USA) was used to digest *PNPLA3*.

**Table 1:** The primer sequences, annealing temperatures, restriction enzymes, and cleavage temperatures used in this study. F–forward; R–reverse.

Target site	Amplicon size (bp)	Primer names and sequences	Annealing temperature	Restriction enzymes	Cleavage temperatures (°C)	References
***PNPLA3 rs738409***	CC 213 GG 129, 93	*PNPLA3*-F (5-CCTGCAGGCAGGAGATGTGT-3)	60°C	NlaIII	37	^55^
		*PNPLA3*-R (5-GCCCTGCTCACTTGGAGAAA -3)				

### Statistical analysis

Sample size calculation was performed assuming a prevalence of MASLD in general population of 25% [56] and 44.5% in H. pylori infected subjects [57]. With 80% of power and alpha level of 0.05, we calculated that at least 194 patients would be needed for the study.

Data were presented as mean ± SD (standard deviation), median IQR (interquartile range), or number of subjects (% of total) as appropriate. Differences between groups were analyzed by Students’ t-test or ANOVA for normal distribution, Wilcoxon rank sum test or Kruskal Wallis test for non-normal distribution. Categorical values were compared using Chi-square tests. The relationship with *H*. *pylori* with MASLD, and with risk of significant-advanced fibrosis (FIB-4 and LSM by VCTE cut-off ≥1.3 and ≥8kPa), were examined by binomial logistic regression model (binary response variable). Univariate models and multiple predictor variable models including model 1) obesity, type-II DBT, hypertriglyceridemia and hypertension; Model 2) obesity, type-II DBT, hypertriglyceridemia, hypertension and rs738409 *PNPLA3* (G-allele) as covariates were assessed. Data were analyzed using SPSS 22.0, and Jamovi 2.5.1. Two-tailed P < 0.05 was considered statistically significant. Manuscript and data followed the STROBE guidelines for cross-sectional studies (S1 Table)

## Results

### Study population

**Fig 1:**
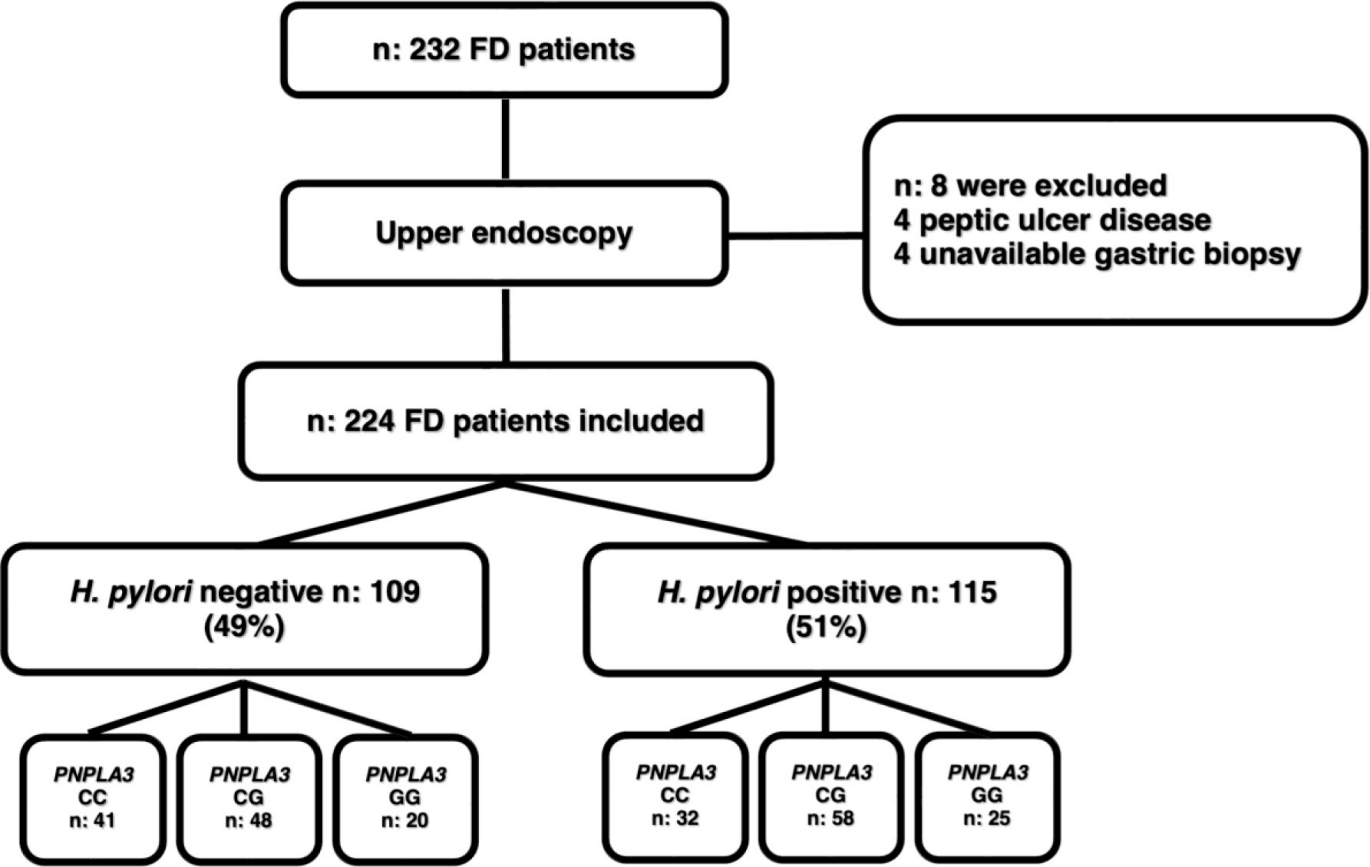
Study flow-chart. Functional dyspepsia (FD), *H*. *pylori* negative, *H*. *pylori* positive, metabolic dysfunction-associated steatotic liver disease (MASLD).

Two hundred and thirty-two patients with FD who met Rome-IV criteria were evaluated before upper endoscopy. Eight patients were excluded, and 224 patients were included for analysis. A flowchart of the study and baseline characteristics of patients appears in Fig 1 and Table 2. The cohort comprised 145 (65%) women and 78 (35%) men with a median age of 52 (IQR, 42–60) years. Regarding clinical comorbidities, 45 (20%) patients had type-II DBT, 77 (34%) had hypertension, and the median BMI was 27.4 (IQR, 24–30) kg/m^2^, with 70 (31%) classified as obese (BMI ≥ 30 kg/m^2^). Additionally, hypercholesterolemia was present in 129 (58%) patients, hypertriglyceridemia in 85 (38%), regular physical activity in 96 (43%) patients, smoking was present in 30 (13%), daily coffee intake in 26 (12%), and moderate alcohol consumption in 82 (37%) subjects. The FD syndromes were as follows: 115 (52%) patients had EPS, 75 (33%) had PDS and 33 (15%) exhibited EPS/PDS overlap. *H*. *pylori*-positive gastric biopsies were detected in 115 (51%) patients. The prevalence of MASLD was 116/224 (52%) (Table 2). The viral serologic status of the study population for HBV and HCV was negative. The frequencies of the PNPLA3 rs738409 alleles were as follows: CC 33%, CG 47% and GG 20%, respectively, and the distribution of the genotypes was in Hardy-Weinberg equilibrium.

**Table 2:** *H*. *pylori* infection, and clinical variables in FD subjects. Data are expressed as median (IQR, interquartile range), mean (SD, standard deviation), or percentage (%) of the total. For parametric continuous variables, Students’ t-test was used. For non-parametric continuous variables, Wilcoxon rank sum test was used. For categorical variables, Chi-square test was used. ns: non-significant.

Variable		Total cohort	*H*. *pylori* (-)	*H*. *pylori* (+)	p
	n (%)	n: 224	n: 109 (49)	115 (51)	
**Age, years**	Median (IQR)	52 (41.5–60)	52 (39.8–61)	52 (43.5–60)	ns
**Gender, female**	n (%)	146 (65)	73 (67)	73 (63)	ns
**PDS**	n (%)	75 (33)	44 (40) *	31 (27)	0.047
**EPS**	n (%)	115 (51)	47 (43)	68 (59) *	0.024
**Overlap**	n (%)	33 (15)	17 (16)	16 (14)	ns
**Izumo scale**	Median (IQR)	10 (7–12)	9 (7–11)	10 (8–12)	ns
**BMI**	Median (IQR)	27.4 (24.6–30.9)	26.7 (24.5–29)	28.7 (24.8–32.6) *	0.005
**BMI ≥ 30**	n (%)	70 (31)	23 (21)	47 (41) *	0.002
**Type-II DBT**	n (%)	45 (20)	20 (18)	25 (22)	ns
**Hypertension**	n (%)	77 (34)	37 (34)	40 (35)	ns
**Triglycerides ≥ 150 mg/dl**	n (%)	43 (19)	20 (22)	21 (18)	ns
**Cholesterol ≥ 200 mg/dL**	n (%)	129 (58)	58 (53)	71 (68)	ns
**Regular physical activity**	n (%)	96 (43)	44 (40)	52 (45)	ns
**Coffee intake**	n (%)	26 (12%)	15 (14%)	11 (10%)	ns
**Smoking**	n (%)	30 (13)	12 (11)	18 (16)	ns
**Moderate alcohol consumption**	n (%)	82 (37)	36 (33)	46 (40)	ns
**MASLD**	n (%)	116 (52)	45 (41)	71 (62) *	0.003
**Ferritin mg/dL**	Median (IQR)	143 (75.4–256)	143 (75.4–231)	145 (75.8–296)	ns
**HDL mg/dL**	Median (IQR)	46.5 (40–56.2)	51 (43–60)	44 (37–51) *	<0.001
**LDL mg/dL**	Median (IQR)	115 (96–142)	111 (91–134)	121 (99–143) *	0.043
**Glucose mg/dL**	Median (IQR)	94 (87–102)	92.5 (88–98)	95 (87–105)	ns
**HOMA**	Median (IQR)	1.6 (0.9–2.8)	1.5 (0.8–2.5)	1.9 (1–3.2)	0.026
**PNPLA3**	CC, n (%)	73 (33)	41 (38)	32 (28)	ns
	CG, n (%)	106 (47)	48 (44)	58 (50)	
	GG, n (%)	45 (20)	20 (18)	25 (28)	

### *H*. *pylori* infection, clinical variables, and MASLD

We first evaluated the association of *H*. *pylori* infection and clinical variables in our cohort. No differences were observed regarding *H*. *pylori* status in age, gender, hypertension, hypercholesterolemia, hypertriglyceridemia, ferritin, glucose, PNPLA3 genotype, regular physical activity, smoking, Izumo scale, coffee intake and moderate alcohol consumption (p>0.05, ns: non-significant) (Table 2). In FD syndromes, *H*. *pylori*-positive status was significantly associated with EPS (p<0.05), while *H*. *pylori*-negative status with PDS (p<0.05). No association at *H*. *pylori* status was observed in overlap EPS/PDS (p: ns). *H*. *pylori* infection was significantly associated with high BMI, obesity, high HOMA index, elevated LDL, low HDL values, and MASLD (p<0.05) (Table 2).

**Fig 2:**
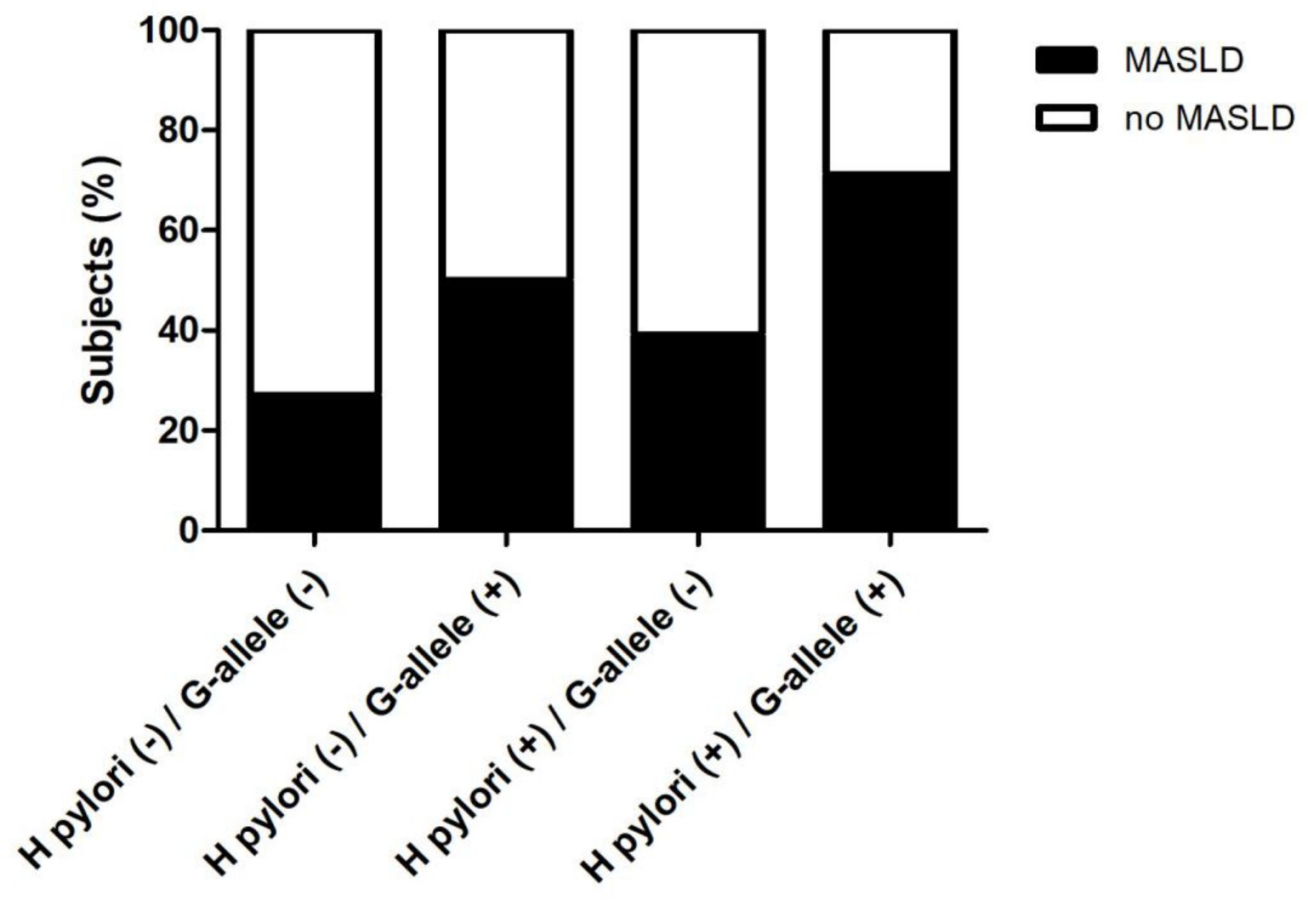
MASLD patients stratified according to *H*. *pylori* status and G-allele PNPLA3. Data are expressed as (%) percentage of subjects. Chi-square test was used. * p < 0.05.

Since, rs738409 PNPLA3 (G-allele), was linked with MASLD in the general population [8], we explored the association of *H*. *pylori* and G-allele PNPLA3 with MASLD in our cohort (Fig 2). *H*. *pylori*-positive subjects harboring G-allele PNPLA3 increased the proportion of MASLD subjects to 71% (p<0.05), compared to *H*. *pylori* positive G-allele-negative (37%), *H*. *pylori*-negative harboring G-allele (50%), and H. pylori negative G-allele-negative (26%) (Fig 2). Likewise, the combination of *H*. *pylori*-negative status and G-allele-negative status was associated with a higher proportion of subjects without MASLD (74%, p<0.05). Moreover, we evaluated by univariate and multivariate binary logistic regression analysis if *H*. *pylori* infection was an independent risk factors for MASLD (Table 3). Univariate analysis indicated that the odds ratio (OR) for MASLD with *H*. *pylori*-positive infection was 2.29 (1.34–3.92; p = 0.002). After adjusting for the most important MASLD risk factors (obesity, type-II DBT, hypertriglyceridemia and hypertension [1]), the OR for MASLD with *H*. *pylori*-positive infection was 1.98 (1.08–3.64; p = 0.028). However, this association became not significant when G-allele PNPLA3 was added to the model (Table 3). Collectively, these data suggest that the association of *H*. *pylori* with MASLD is influenced by the prevalence of G-allele PNPLA3, and together may synergize to increase the risk of MASLD.

**Table 3:** Univariate and multivariate binary logistic regression of *H*. *pylori* infection and MASLD. Data are expressed as median (IQR, interquartile range), or percentage (%) of total. ns: non-significant.

		Univariate		Adjusted for Obesity, Type-II DBT, Hypertriglyceridemia and Hypertension		Adjusted for Obesity, Type-II DBT, Hypertriglyceridemia, Hypertension and G-allele PNPLA3	
	**MASLD, n (%)**	**OR (95% CI)**	***p***	**OR (95% CI)**	***p***	**OR (95% CI)**	***p***
***H*. *pylori* (-)**	***45 (41)***						
***H*. *pylori* (+)**	***71 (62)***	**2.29 (1.34–3.92)**	**0.002**	**1.98 (1.08–3.64)**	**0.028**	**1.86 (0.98–3.51)**	**ns**

### *H*. *pylori* infection, markers of liver injury, and fibrosis in MASLD subjects

**Fig 3:**
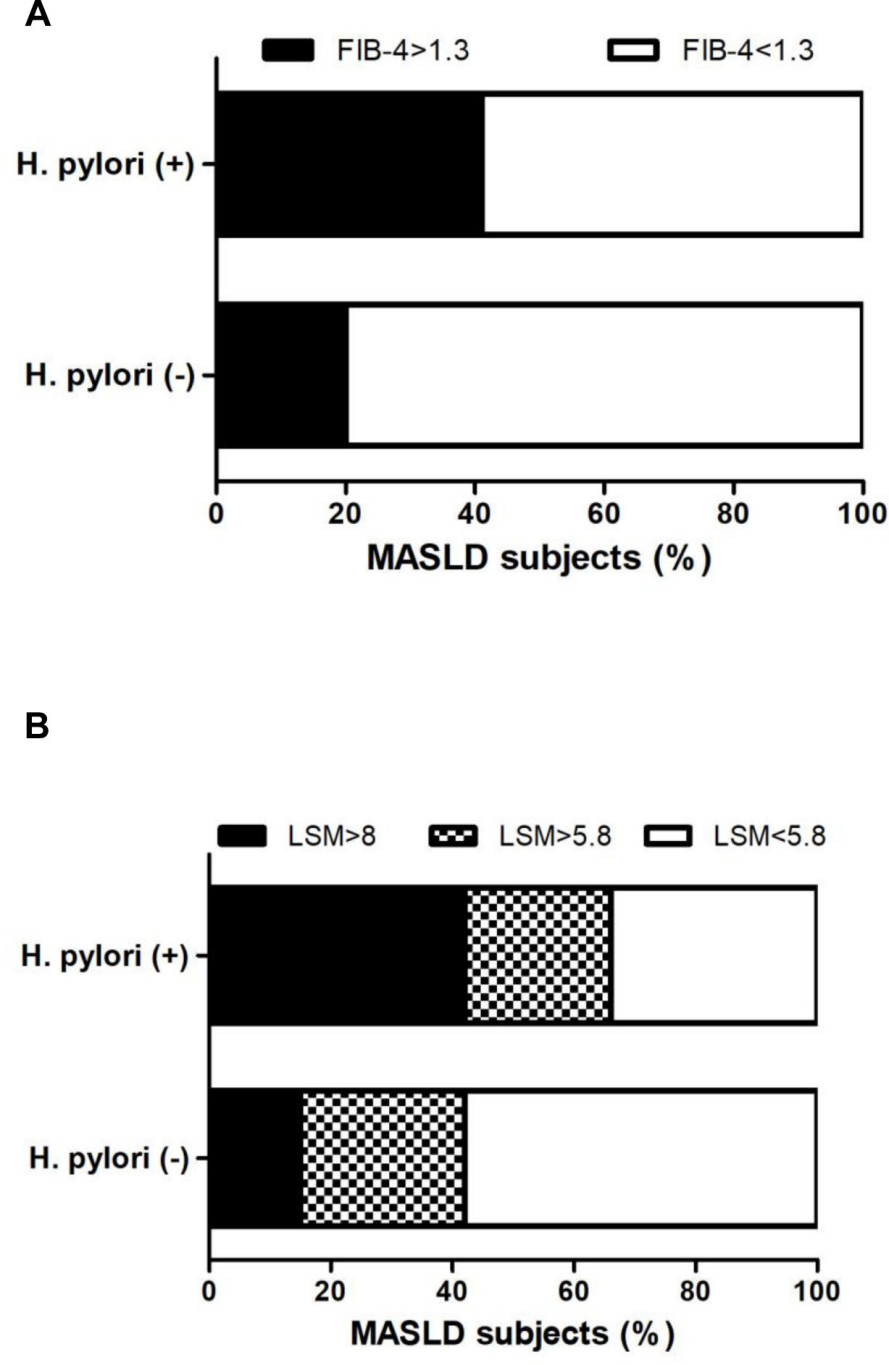
Non-invasive markers of high risk of significant/advanced fibrosis in patients with MASLD stratified according to *H*. *pylor*i status. A) FIB-4 score ≥ 1.3 or <1.3. B) Liver stiffness measurement by vibration controlled transient elastography (LSM by VCTE), stratified by LSM cut-offs to rule-out significant fibrosis < 5.8 kPa, intermediate risk ≥5.8 kPa-<8 kPa, and to rule-out advanced fibrosis ≥8 kPa [50–52]. Data are expressed as (%) percentage of MASLD subjects. Chi-square test was used. * p < 0.05.

Next, we evaluated the influence of *H*. *pylori* infection on non-invasive markers of liver injury and fibrosis in our cohort of MASLD subjects (n: 116) (Table 4). *H*. *pylori*-positive status showed no difference in total bilirubin, ALT, ALP, GGT, platelets, albumin, ferritin and PNPLA3 genotype. Serum AST levels were significantly increase in *H*. *pylori*-positive subjects (p<0.05) (Table 4). To further evaluate liver injury, non-invasive markers of fibrosis—FIB-4 score and liver stiffness measurement (LSM) by vibration control transient elastography (VCTE)—was determined. *H*. *pylori*-positive status showed no difference in FIB-4 (Table 4). LSM by VCTE was significantly increased in *H*. *pylori* infection (p<0.05) (Table 4). Furthermore, using cut-off values to rule-out significant/advanced fibrosis (FIB-4 ≥1.3, LSM ≥5.8 kPa and LSM ≥8 kPa), we observed a significant trend of higher proportion of *H*. *pylori*-positive subjects with FIB-4 ≥1.3 (44%, p 0.04), LSM ≥5.8 kPA (66%, p 0.027), and LSM ≥8 kPa (39%, p 0.013) compared to *H*. *pylori*-negative patients (FIB-4 ≥1.3 21%; LSM ≥5.8 kPa 40%, LSM ≥8 kPa 13%) (Fig 3A and 3B).

**Table 4:** *H*. *pylori* infection, non-invasive markers of liver injury and fibrosis in MASLD subjects. Data are expressed as median (IQR, interquartile range), or percentage (%) of total. For parametric continuous variables, Students’ t-test was used. For non-parametric continuous variables, Wilcoxon rank sum was used. For categorical variables, Chi-square test was used. ns: non-significant.

Variable		H. pylori (-)	H. pylori (+)	p
	n (%)	45 (39)	71 (61)	
**Age, years**	Median (IQR)	52 (42–59)	53 (48.5–60)	ns
**Gender, female**	n (%)	27 (60%)	39 (55%)	ns
**BMI**	Median (IQR)	27.6 (26.8–31.1)	31.2 (27.4–34.1)	ns
**BMI ≥ 30**	n (%)	17 (38)	41 (58%)	0.036
**Type-II DBT**	n (%)	15 (33)	23 (32)	ns
**HOMA**	Median (IQR)	2.5 (1.5–3.33)	2.8 (1.9–3.7)	ns
**Hypertension**	n (%)	17 (38%)	29 (41%)	ns
**Triglycerides ≥ 150 mg/dl**	n (%)	13 (29%)	20 (28%)	ns
**Total bilirubin, mg/dL**	Median (IQR)	0.6 (0.5–0.9)	0.7 (0.6–1)	ns
**AST, IU/L**	Median (IQR)	21 (18–29)	28.0 (20–42)*	0.047
**ALT, IU/L**	Median (IQR)	27 (17–43)	31 (21–48) *	ns
**ALP, IU/L**	Median (IQR)	84 (72–110)	96 (75–119)	ns
**GGT, IU/L**	Median (IQR)	33 (22–44)	39 (26–50)	ns
**FIB4**	Median (IQR)	0.9 (0.8–1.3)	1.1 (0.9–1.7)	ns
Platelets, 10^9^/L	Mean (SD)	235 (61)	236 (74)	ns
**Albumin, mg/dL**	Median (IQR)	4.1 (3.9–4.4)	4.0 (3.9–4.2)	ns
**Ferritin, mg/dL**	Median (IQR)	219 (115–304)	178.0 (96–350)	ns
**LSM, kPa**	Median (IQR)	5 (4.2–7.2)	6.8 (4.9–8.8)*	0.015
**PNPLA3**	CC, n (%)	10 (27.0)	8 (13.1)	ns
	CG, n (%)	16 (43.2)	32 (52.5)	
	GG, n (%)	11 (29.7)	21 (34.4)	

**Fig 4:**
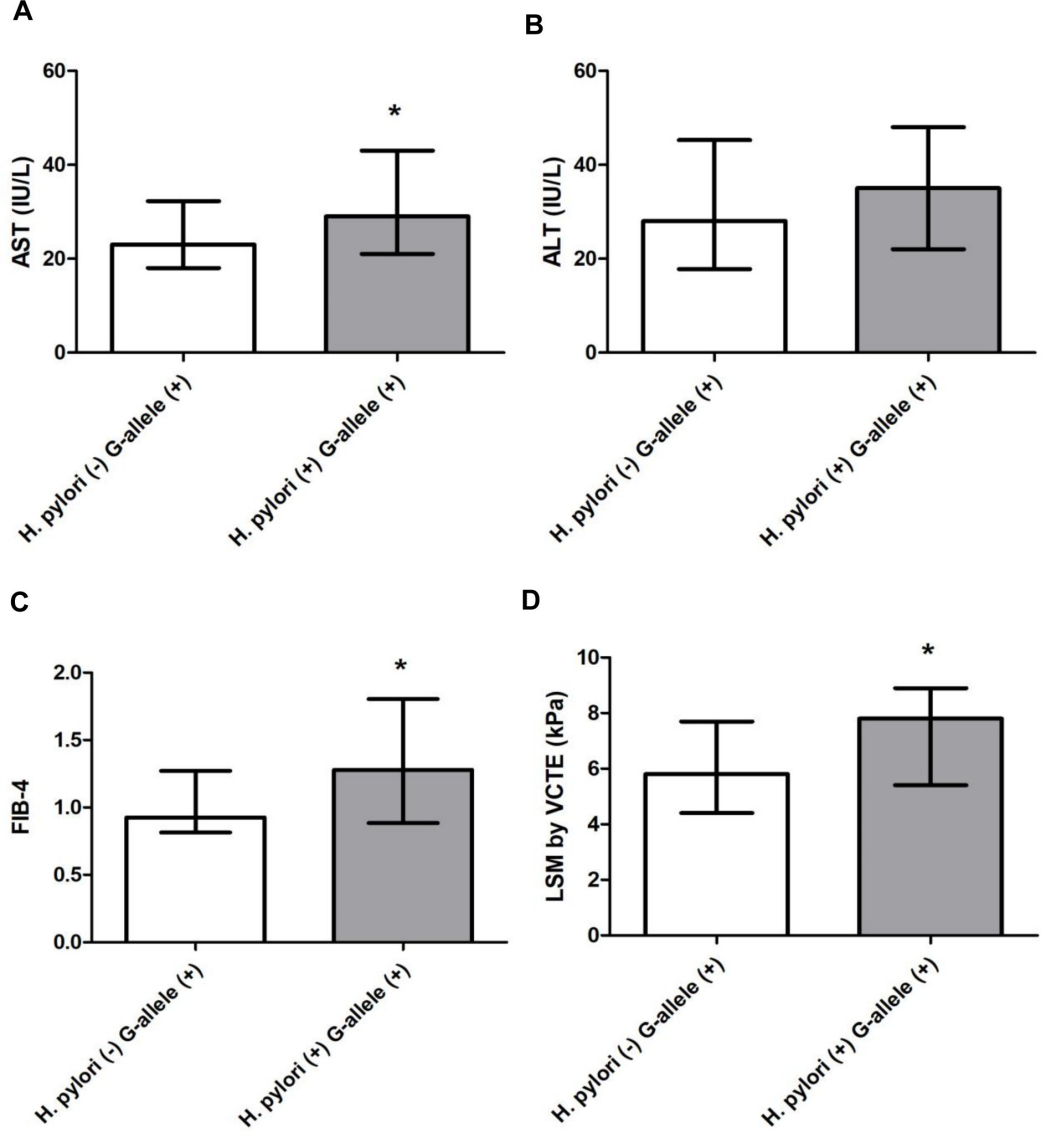
Non-invasive markers injury and fibrosis in patients with MASLD harboring G-allele PNPLA3 stratified according to *H*. *pylori* status. A) AST values, UI/L. B) ALT values, IU/L. C) FIB-4. D) Liver stiffness measurement by vibration controlled transient elastography (LSM by VCTE), kPa. Data are expressed as median (IQR, interquartile range). Wilcoxon rank sum test was used. * p < 0.05.

**Fig 5:**
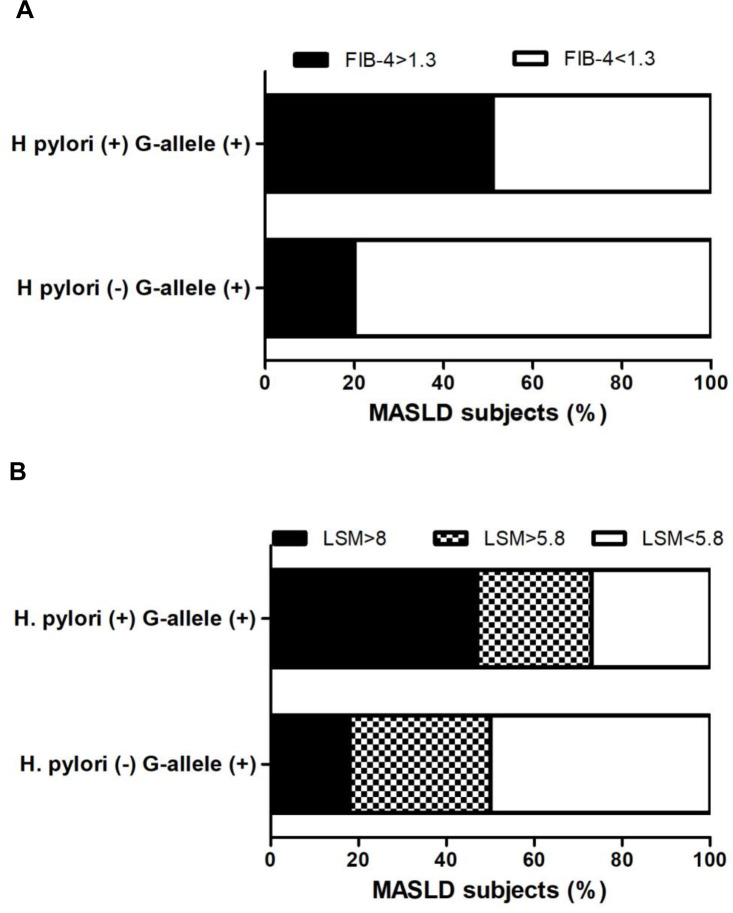
Non-invasive markers of high risk of significant/advanced fibrosis in patients with MASLD harboring G-allele PNPLA3 stratified according to *H*. *pylori* status. A) FIB-4 score ≥ 1.3 or <1.3. B) Liver stiffness measurement by vibration controlled transient elastography (LSM by VCTE), stratified by LSM cut-offs to rule-out significant fibrosis < 5.8 kPa, intermediate risk ≥5.8 kPa-<8 kPa, and to rule-out advanced fibrosis ≥8 kPa [50–52]. Data are expressed as (%) percentage of MASLD subjects. Chi-square test was used.

Then, we assessed the association with *H*. *pylori* and G-allele PNPLA3 with non-invasive markers of liver fibrosis, focusing on the MASLD G-allele PNPLA3-positive cohort (n: 92/116, 79%). *H*. *pylori* infection was significantly associated with elevated AST (p <0.05), but no difference was observed at ALT values (p ns) (Fig 4A and 4B). Non-invasive markers of liver fibrosis, FIB-4, and LSM by VCTE, were increased in *H*. *pylori*-positive patients (p <0.05) (Fig 4C and 4D). Likewise, *H*. *pylori* infection was associated with a higher proportion of patients with FIB-4 ≥1.3 (49%, p <0.001), LSM ≥ 5.8 kPa (73%, p 0.0013) and LSM ≥ 8 kPa (47%, p <0.001) (Fig 5A and 5B). Moreover, we assessed by logistic regression model the impact of *H*. *pylori* status with FIB-4 ≥1.3 and LSM ≥ 8 kPa (Table 5). The OR for FIB-4 ≥1.3 and LSM ≥ 8 kPa with *H*. *pylori* infection was 2.76 (1.16–6.59; p = 0.022) and 3.97 (1.56–10.1; p = 0.004), respectively. After adjusting for the most clinical relevant MASLD risk factors for fibrosis (hypertension, hypertriglyceridemia, obesity and type-II DBT [1]), the OR for FIB-4 ≥1.3 and LSM ≥ 8 kPa with *H*. *pylori*-positive infection was 3.41 (1.28–9.01; p = 0.014) and 4.59 (1.55–13.65; p = 0.006), respectively. Remarkably, when G-allele PNPLA3 was added to the model, this association remain unchanged (Table 5). Collectively, these data suggest that *H*. *pylori* infection is associated with markers of liver fibrosis and may increase the risk of significant/advanced fibrosis in MASLD subjects.

**Table 5:** Univariate and multivariate binary logistic regression of *H*. *pylori* infection with FIB-4 >1.3 and LSM ≥ 8kPa. OR: odds ratio, CI: confidence interval, ns: non-significant.

		Univariate		Adjusted for Hypertension, Hypertriglyceridemia, Obesity and Type-II DBT		Adjusted for Hypertension, Hypertriglyceridemia, Obesity, Type-II DBT and G-allele PNPLA3	
		**OR (95% CI)**	***p***	**OR (95% CI)**	***p***	**OR (95% CI)**	***p***
**MASLD LSM >8kPa, n (%)**							
***H*. *pylori* (-)**	***7 (16)***						
***H*. *pylori* (+)**	***30 (42)***	**3.97 (1.56–10.1)**	**0.004**	**4.59 (1.55–13.65)**	**0.006**	**3.92 (1.31–11.71)**	**0.0015**
**MASLD FIB-4 >1.3, n (%)**							
***H*. *pylori* (-)**	***9 (20)***						
***H*. *pylori* (+)**	***29 (41)***	**2.76 (1.16–6.59)**	**0.022**	**3.41 (1.28–9.01)**	**0.014**	**2.98 (1.11–8.13)**	**0.031**

### *H*. *pylori* infection, MASH, and fibrosis in biopsy-proven MASLD subjects

**Fig 6:**
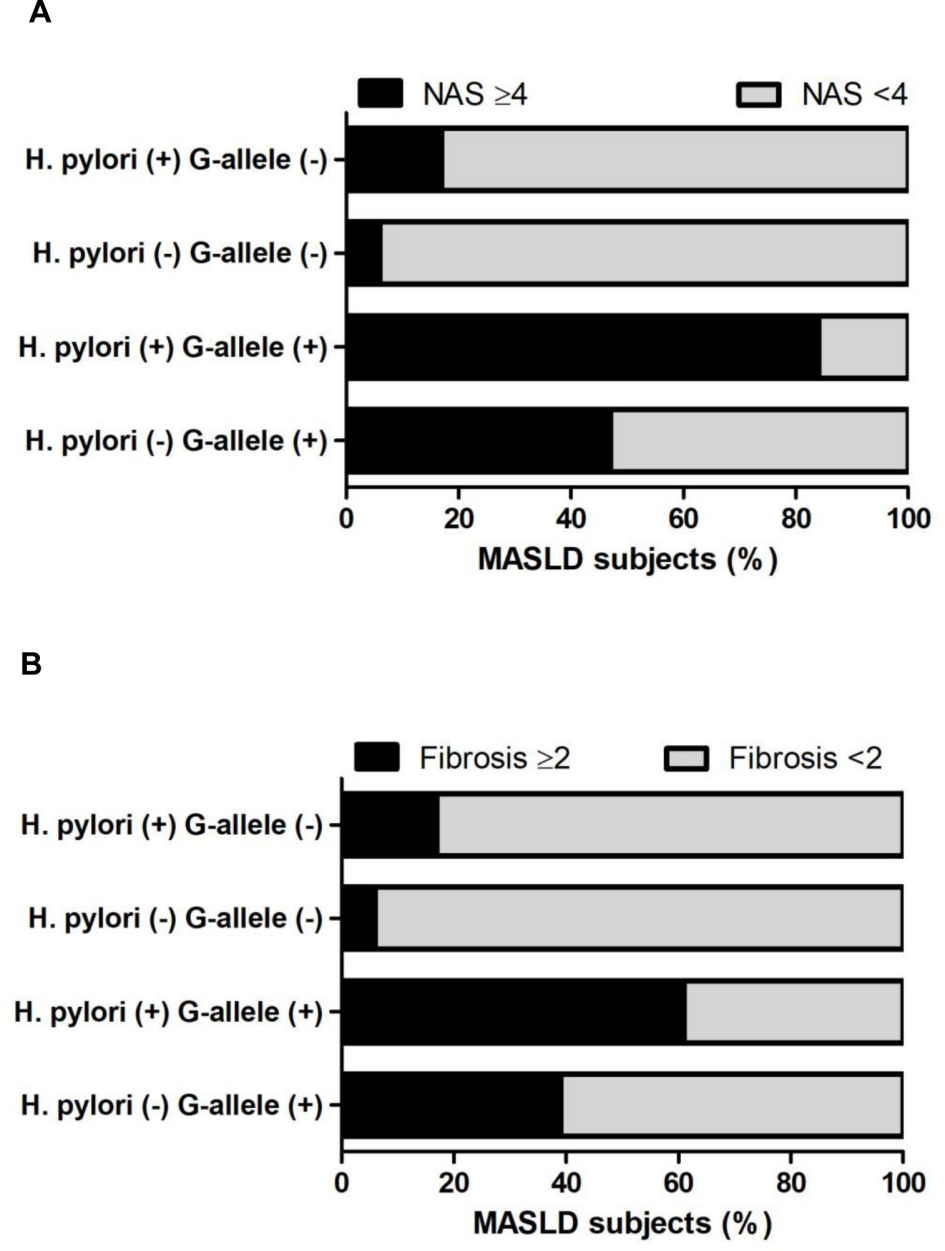
Metabolic dysfunction-associated steatohepatitis (MASH) (NAS score ≥ 4), and significant/advanced fibrosis (Fibrosis ≥ 2) in patients with biopsy-proven MASLD stratified according to *H*. *pylori* status and G-allele PNPLA3. A) NAS score ≥4 or <4. B) Fibrosis stage ≥ 2 or <2. Data are expressed as (%) percentage of MASLD subjects. Chi-square test was used.

To more directly address the association of *H*. *pylori* infection with liver inflammation and fibrosis, we next evaluated MASLD histological severity by NAS score and fibrosis stage in a cohort of biopsy-proven MASLD (Table 6). The cohort comprise 101 patients with biopsy-proven MASLD, with a median age of 54 years (IQR, 47–62), 41% female, with a mean BMI of 31.2 (SD, 4), and *H*. *pylori*-positive infection were detected in 37 out of 101 (36.6%) patients. In this cohort, *H*. *pylori*-positive infection was significantly associated with higher GGT and LSM (p<0.05) (Table 6). Histopathology from *H*. *pylori*-positive infection patients displayed increased ballooning grade, NAS score, and higher proportion of MASH subjects. Moreover, a significant association with *H*. *pylori*-positive infection and increased fibrosis stage was also observed (Table 6). Then, we focused on the interplay between *H*. *pylori* infection and G-allele PNPLA3 with severity of MASLD histology (Fig 6). *H*. *pylori*-positive infection with G-allele PNPLA3 was significantly associated with higher proportion of patient with MASH (84%), versus *H*. *pylori*-negative status with G-allele PNPLA3 (47%), *H*. *pylori*-positive infection without G-allele PNPLA3 (17%), and *H*. *pylori*-negative status without G-allele PNPLA3 (6%) (p<0.001) (Fig 6A). Furthermore, consistent with our observation by non-invasive test in the FD-MASLD cohort, the presence of significant/advanced fibrosis (Fibrosis stage ≥2) was significantly increased in *H*. *pylori*-positive infection with G-allele PNPLA3 (61%), versus in *H*. *pylori*-negative infection with G-allele PNPLA3 (39%), *H*. *pylori*-positive infection without G-allele PNPLA3 (17%), and *H*. *pylori*-negative status without G-allele PNPLA3 (6%) (p: 0.001) (Fig 6B). Collectively, these data suggest that *H*. *pylori* infection is associated MASLD histological severity and fibrosis. Moreover, the combination of *H*. *pylori* infection and G-allele PNPLA3 seems to increase MASLD severity higher than each trait alone.

**Table 6:** *H*. *pylori* infection, and clinical variables in biopsy-proven MASLD subjects. Data are expressed as median (IQR, interquartile range), mean (SD, standard deviation), or percentage (%) of the total. For parametric continuous variables, Students’ t-test was used. For non-parametric continuous variables, Wilcoxon rank sum test was used. For categorical variables, Chi-square test was used. ns: non-significant.

Variable		Total cohort	H. pylori (-)	H. pylori (+)	p
	n (%)	n: 101	n: 64 (63)	37 (37)	
**Age, years**	Median (IQR)	54 (47–62)	56.5 (47–62)	53 (47.2–60)	ns
**Gender, female**	n (%)	41 (41)	27 (42)	14 (38)	ns
**BMI**	Mean (SD)	31.2 (4)	30.6 (3.5)	32.1 (4.7)	ns
**BMI ≥ 30**	n (%)	59 (58)	36 (56)	23 (62)	ns
**Type-II DBT**	n (%)	65 (64)	41 (64)	24 (65)	ns
**HOMA**	Median (IQR)	3.5 (2.3–4.9)	3.2 (2.3–4.7)	3.8 (2.2–5.6)	ns
**Hypertension**	n (%)	50 (49)	31 (48)	19 (51)	ns
**Triglycerides ≥ 150 mg/dl**	n (%)	48 (47)	34 (53)	14 (38)	ns
**LSM, kPa**	Median (IQR)	7.5 (6–10.9)	7 (5.5–9.1)	8.1 (6.8–17.9)	0.013
**AST IU/L**	Median (IQR)	30 (23–44)	30 (23–41)	35 (21–49)	ns
**ALT IU/L**	Median (IQR)	36 (26–53)	35.5 (26–51)	36 (32–53)	ns
**FAL IU/L**	Median (IQR)	89 (76–119)	85 (73–145)	99 (82–118)	ns
**GGT IU/L**	Median (IQR)	45 (29–76)	41 (28–65)	56 (36–91)	0.037
**Ferritin mg/dL**	Median (IQR)	311 (142–539)	348-161-551)	265 (135–496)	ns
**Platelets, 109/L**	Mean (SD)	214 (65)	220 (62)	202 (69)	ns
**PNPLA3**	CC, n (%)	24 (24)	18 (28)	6 (16)	ns
	CG, n (%)	51 (50)	34 (53)	17 (46)	
	GG, n (%)	26 (26)	12 (19)	14 (38)	
**Liver Histology**					
**Steatosis Grade**	1, n (%)	44 (43.6)	27 (42.2)	17 (45.9)	ns
	2, n (%)	52 (51.5)	35 (54.7)	17 (45.9)	
	3, n (%)	5 (5)	2 (3.1)	3 (8.1)	
**Lobular Inflammation Grade**	0, n (%)	6 (5.9)	6 (9.4)	0 (0)	ns
	1, n (%)	80 (79.2)	50 (78.1)	30 (81.1)	
	2, n (%)	15 (14.9)	8 (12.5)	7 (18.9)	
**Ballooning Grade**	0, n (%)	31 (30.7)	27 (42.2)	4 (10.8)	<0.001
	1, n (%)	50 (49.5)	33 (51.6)	17 (45.9)	
	2, n (%)	20 (19.8)	4 (6.2)	16 (43.2)	
**NAS Score**	1, n (%)	2 (2)	2 (3.1)	0 (0.0)	0.01
	2, n (%)	12 (11.9)	11 (17.2)	1 (2.7)	
	3, n (%)	37 (36.6)	28 (43.8)	9 (24.3)	
	4, n (%)	29 (28.7)	15 (23.4)	14 (37.8)	
	5, n (%)	16 (15.8)	6 (9.4)	10 (27)	
	6, n (%)	5 (5)	2 (3.1)	3 (8.1)	
**MASH (NAS ≥ 4)**	0, n (%)	51 (50.5)	41 (64.1)	10 (27)	0.001
	1, n (%)	50 (49.5)	23 (35.9)	27 (73)	
**Fibrosis Stage**	0, n (%)	19 (18.8)	14 (21.9)	5 (13.5)	0.003
	1, n (%)	43 (42.6)	31 (48.4)	12 (32.4)	
	2, n (%)	15 (14.9)	11 (17.2)	4 (10.8)	
	3, n (%)	6 (5.9)	4 (6.2)	2 (5.4)	
	4, n (%)	18 (17.8)	4 (6.2)	14 (37.8)	

## Discussion

The principal findings of this study pertain to the potential association of *H*. *pylori* with MASLD severity. Our results suggest that *H*. *pylori* infection is associated with: (i) cardiometabolic risk factors for MASLD, (ii) steatotic liver phenotype influenced by the prevalence of G-allele *PNPLA3*, (iii) increased AST and LSM by VCTE in MASLD subjects, (iv) an independent risk of significant/advanced fibrosis by FIB-4 and LSM in MASLD subjects, (v) increased NAS score and fibrosis stage, and (vi) the combination of *H*. *pylori* infection and the G-allele PNPLA3 genotype appeared to exacerbate MASLD severity, indicating a synergistic effect.

Ethiopathogenic association between *H*. *pylori* infection and MASLD is a matter of debate that was intended to be answered by various researcher worldwide from the bench and clinical stand point [34, 58]. It is important to note that murine models of *H*. *pylori* infection do not have a fatty liver phenotype, but under a high-fat diet, *H*. *pylori*-positive mice had more hepatic steatosis and release of inflammatory mediators [59]. In patients with *H*. *pylori* infection, an unfavorable cardiometabolic lipid profile featured by high triglycerides, total cholesterol and LDL-C and decreased HDL-C levels were displayed [32]. Our study showed a correlation with *H*. *pylori* infection with cardiometabolic risk factors and steatotic liver by ultrasound. Since the pathophysiology of MASLD is only partially revealed, it is considered as a multifactorial disorder, attributed to multiple or parallel “hits”, both genetic and environmental [6]. In this regard, we evaluated the association of an environmental risk factor like *H*. *pylori* infection and the rs738409 G-allele *PNPLA3*, the most important genetic variant associated with MASLD. We observed that *H*. *pylori*-infected subjects harboring G-allele PNPLA3 dramatically increased the proportion of MASLD subjects to 71%, and when *H*. *pylori* and G-allele both were negative. a higher proportion of subjects without MASLD (74%) was detected. Then, by multivariate analysis, we revealed that the association of *H*. *pylori* with MASLD is influenced by the prevalence of G-allele PNPLA3. Based on this observation, *H*. *pylori* infection may facilitate some metabolic derangements that predispose to steatotic liver phenotype in subjects harboring G-allele *PNPLA3*.

Liver fibrosis is the most relevant prognostic factor in patients with MASLD [60]. Previous studies on murine models showed that *H*. *pylori*-infected mice had increased markers of liver injury and fibrosis compared to uninfected mice [61]. A clinical study in morbidly obese patients demonstrated, on histopathological approach, that *H*. *pylori* infection increased NAS score and fibrosis [62]. Also, in a recent study form China using serological test for *H*. *pylori* diagnosis, ultrasound attenuation parameter (UAP) for diagnosis of MASLD, and transient elastography using FibroTouch, observed that *H*. *pylori* infection is a risk factor for increased liver stiffness [57]. The precise mechanisms underlying the connection between gastric *H*. *pylori* infection and extra-gastroduodenal diseases remain unclear. For instance, local inflammation in the gastric and duodenal mucosa may lead to release of proinflammatory cytokines that increase gut permeability and access to portal circulation [32]. In this regard, *H*. *pylori* and other bacteria and toxins as well as cytokines may directly affect the hepatic parenchyma leading to and inflammatory milieu that may activate stellate cells to generate fibrosis [32]. Our study showed a correlation with *H*. *pylori*-infection with elevated non-invasive markers of liver fibrosis by FIB-4 score and LSM by VCTE. Our observation was independent of risk factors of disease severity such as type-II DBT, obesity and G-allele PNPLA3. Noteworthy, this observation was further confirmed in the biopsy-proven MASLD cohort, and appear to have a synergistic effect with G-allele PNPLA3 exacerbating MASLD severity. Confirmation from mechanistic studies is needed to clarify the clinical role of *H*. *pylori* infection and fibrogenesis in MASLD. In this regard, the interaction between host genetic polymorphism background, *H*. *pylori* virulence genes and low-grade duodenal inflammation could explain high-risk of fibrosis in MASLD, and will be evaluated in our future research. It is beyond the scope of this study to evaluate the impact of *H*. *pylori* eradication on MASLD markers of liver injury and fibrosis. However, information is available from four clinical controlled studies [33, 63–65]. While this four studies had disparate design, follow-up, and end points making it difficult to compare, it seems that eradication therapy as adjunct to lifestyle intervention may improve non-invasive markers of steatosis, fibrosis and inflammation [33, 63–65]. Therefore, whether *H*. *pylori* eradication influences the progression of MASLD is still controversial and needs to be confirmed by multicentric studies in the future.

The strengths of this study are that it is a prospective evaluation from a well-defined FD-MASLD cohort. Endoscopists and Pathologists were blinded to medical history before evaluation. Non-invasive marker of liver fibrosis, FIB-4, and LSM by VCTE were performed in all subjects. We choose FD patients for the current study because gastric biopsy samples are mostly available, which is the gold standard for *H*. *pylori* infection diagnosis [66]. Also, FD etiopathogenesis is not related to MASLD and may not increase selection bias of the cohort [7, 17]. In the retrospective cohort of biopsy-proven MASLD, active *H*. *pylori* gastric infection and PNPLA3 genotype were performed in all subjects.

The limitations of the study include: (1) population studied: Our cohort comprised of South-American population of functional dyspepsia. Though we tried to investigate a cohort whose characteristics could resembled those of the general population, the modality of cohort recruitment did not allow us to affirm that our cohort was fully representative of the general population. However, our cohort was recruited independently from the hypothesis concerning the high risk of MASLD severity. (2) Based on the non-invasive nature of the study design in FD-cohort, hepatic steatosis was detected by ultrasonography and fibrosis by FIB-4 score and LSM by VCTE but not liver histology, so the absence of histologic data prevents us from reporting the exact prevalence of steatohepatitis and advanced fibrosis. Noteworthy, LSM by VCTE is the most validated non-invasive method to accurately screen fibrosis in MASLD and can predict the occurrence of liver-related events in MASLD [43, 67]. (3) The retrospective nature of the biopsy-proven MASLD cohort renders it susceptible to recall bias, the duration of *H*. *pylori* gastric infection is unknown, and the sample size could be small, but sufficient to show robust statistical significance in major MASLD-histological endpoints.

In conclusion, the results of the current study suggest that *H*. *pylori* infection may play a role in the progression of liver injury and fibrosis in patients with MASLD, particularly in those with specific genetic predispositions like G-allele PNPLA3, highlighting the importance of further investigation into the underlying mechanisms and potential therapeutic implications.

## Supporting information

S1 Table Checklist STROBE statement—Checklist of items that should be included in reports of observational studies. (DOC)

## Abbreviations

ALP: alkaline phosphatase
ALT: alanine aminotransferase
AST: aspartate aminotransferase
BMI: body mass index
cagA: cytotoxin associated genes A
CI: confidence interval
DBT: Diabetes Mellitus
EPS: epigastric pain syndrome
FD: functional dyspepsia
GGT: gamma-glutamyl transferase
H. pylori: Helicobacter pylori
IQR: interquartile rang
kPa: kilopasca
LSM: : liver stiffness measurement
NSAID: non-steroidal anti-inflammatory drugs
OR: odds ratio
PNPLA3: patatin like phospholipase domain containing 3
PCR: polymerase chain reaction
PDS: postprandial distress syndrome
PPI: proton pump inhibitors
SD: standard deviation
VCTE: vibration controlled transient elastography
