# Supplementary material for: The interplay between Helicobacter pylori infection and rs738409 PNPLA3 in metabolic dysfunction-associated steatotic liver disease
Source: PLoS One. 2024 Sep 23;19(9):e0310361. doi: 10.1371/journal.pone.0310361 (PMC11419387; doi:10.1371/journal.pone.0310361)
Supplement: S1 Table — (DOC) [file pone.0310361.s001.doc]

**STROBE Statement**

Checklist of items that should be included in reports of observational studies

| **Section/Topic** | Item No | Recommendation | Reported on Page No | Relevant text from Manuscript |
| --- | --- | --- | --- | --- |
| **Title and abstract** | 1 | (*a*) Indicate the study’s design with a commonly used term in the title or the abstract | Page 3 | “A multi-center cross-sectional study was conducted” |
|  |  | (*b*) Provide in the abstract an informative and balanced summary of what was done and what was found | Page 3 | “We aim to evaluate the association of H. pylori and G-allele PNPLA3 in MASLD diagnosis, and markers of severity  ” the combination of H. pylori infection and G-allele PNPLA3 appeared to exacerbate MASLD severity beyond individual effects” |
| Introduction | | | |  |
| Background/rationale | 2 | Explain the scientific background and rationale for the investigation being reported | Page 4-5 | “, we can consider that H. pylori infection may exacerbate the effects of the PNPLA3 rs738409 variant on hepatic milieu, thereby increasing the risk and severity of MASLD.” |
| Objectives | 3 | State specific objectives, including any prespecified hypotheses | Page 5 | “our aim was to evaluate the association of H. pylori and rs738409 G-allele in the PNPLA3 in MASLD severity” |
| Methods | | | |  |
| Study design | 4 | Present key elements of study design early in the paper | Page 5-9 | “In this cross-sectional study, we consecutively recruited patients newly diagnosed with FD symptoms based on Rome-IV criteria who underwent upper endoscopy…” |
| Setting | 5 | Describe the setting, locations, and relevant dates, including periods of recruitment, exposure, follow-up, and data collection | Page 5-9 | “… who underwent upper endoscopy at IOT Medical Center (Posadas, Province of Misiones) and University Hospital San Juan Bautista (Santo Tomé, Province of Corrientes), Argentina, were evaluated in this study. The recruitment period spanned from December 14th 2021 to December 15th 2023.” |
| Participants | 6 | (*a*) *Cohort study*—Give the eligibility criteria, and the sources and methods of selection of participants. Describe methods of follow-up  *Case-control study*—Give the eligibility criteria, and the sources and methods of case ascertainment and control selection. Give the rationale for the choice of cases and controls  *Cross-sectional study*—Give the eligibility criteria, and the sources and methods of selection of participants | Page 5-9 | “The criteria for inclusion were: (1) age between 18 and 70 years, (2) symptoms meeting Rome-IV criteria. The criteria for exclusion before gastroscopy were: (1) progressive, severe diseases requiring active medical management (e.g. uncontrolled diabetes, congestive heart failure, end-stage renal failure, neurological disease, advanced cancer, or psychiatric disorder), (2) those with known causes of chronic liver diseases and significant alcohol consumption (defined as  ≥ 140 g/week for women and  ≥ 210 g/week for men), (3) autoimmune medical conditions (inflammatory bowel disease, celiac disease, vasculitis, connective tissue disease), (4) patients who had taken steatogenic medications (corticosteroids, tamoxifen, amiodarone, methotrexate, amiodarone), (5) patients who had taken antibiotic within the past 3 weeks and (7) history gastric or bariatric surgery. Patients receiving proton pump inhibitors (PPI), H2-blockers or non-steroidal anti-inflammatory drugs (NSAID) were advised to suspend them 14 days before endoscopy.” |
|  |  | (*b*) *Cohort study*—For matched studies, give matching criteria and number of exposed and unexposed  *Case-control study*—For matched studies, give matching criteria and the number of controls per case | Not applicable |  |
| Variables | 7 | Clearly define all outcomes, exposures, predictors, potential confounders, and effect modifiers. Give diagnostic criteria, if applicable | Page 7-10 | “FD patients satisfied the Rome-IV criteria”  “the diagnosis of MASLD required the following: (1) hepatic steatosis detected by ultrasonography; (2) no significant alcohol consumption (defined as <140 g/week for women and <210 g/d for men); (3) the presence of one cardiometabolic risk factor; and (4) no other discernible cause of steatosis.”  “The presence of H. pylori was assessed on gastric biopsies using Giemsa staining”  “risk of significant-advanced fibrosis (FIB-4 and LSM by VCTE cut-off ≥1.3 and ≥8kPa)” |
| Data sources/measurement | 8* | For each variable of interest, give sources of data and details of methods of assessment (measurement). Describe comparability of assessment methods if there is more than one group | Page 6-10 | Described throughout the Materials and Methods section |
| Bias | 9 | Describe any efforts to address potential sources of bias | Page 6-10 | Described throughout the Materials and Methods section |
| Study size | 10 | Explain how the study size was arrived at | Page 10 | “Sample size calculation was performed assuming a prevalence of MASLD in general population of 25% 56 and 44.5% in H. pylori infected subjects 57. With 80% of power and alpha level of 0.05, we calculated that at least 194 patients would be needed for the study” |
| Quantitative variables | 11 | Explain how quantitative variables were handled in the analyses. If applicable, describe which groupings were chosen and why | Page 10 | Details on variables are described in “Statistical Analysis” section |
| Statistical methods | 12 | (*a*) Describe all statistical methods, including those used to control for confounding | Page 10 | Details on statistical methods are described in “Statistical Analysis” section |
|  |  | (*b*) Describe any methods used to examine subgroups and interactions | Page 10 | Details on variables are described in “Statistical Analysis” section |
|  |  | (*c*) Explain how missing data were addressed | Page 6 | Patients with missing gastric biopsies were excluded |
|  |  | (*d*) *Cohort study*—If applicable, explain how loss to follow-up was addressed  *Case-control study*—If applicable, explain how matching of cases and controls was addressed  *Cross-sectional study*—If applicable, describe analytical methods taking account of sampling strategy | Not applicable |  |
|  |  | (*e*) Describe any sensitivity analyses | Page 10 | Details on statistical methods are described in “Statistical Analysis” section |
| Results | | | |  |
| Participants | 13* | (a) Report numbers of individuals at each stage of study—eg numbers potentially eligible, examined for eligibility, confirmed eligible, included in the study, completing follow-up, and analysed | Page 10-11 | Figure 1, Table 2, Figure 2 |
|  |  | (b) Give reasons for non-participation at each stage | Page 10-11 | Figure 1, Table 2 |
|  |  | (c) Consider use of a flow diagram | Page 10-11 | Figure 1 |
| Descriptive data | 14* | (a) Give characteristics of study participants (eg demographic, clinical, social) and information on exposures and potential confounders | Page 10-11 | Table2 |
|  |  | (b) Indicate number of participants with missing data for each variable of interest | Page 10-11 | Figure 1 |
|  |  | (c) *Cohort study*—Summarise follow-up time (eg, average and total amount) |  |  |
| Outcome data | 15* | *Cohort study*—Report numbers of outcome events or summary measures over time |  |  |
|  |  | *Case-control study—*Report numbers in each exposure category, or summary measures of exposure |  |  |
|  |  | *Cross-sectional study—*Report numbers of outcome events or summary measures | Page 10-13 | Table 2, Table 6 |
| Main results | 16 | (*a*) Give unadjusted estimates and, if applicable, confounder-adjusted estimates and their precision (eg, 95% confidence interval). Make clear which confounders were adjusted for and why they were included | Page 10-14 | Table 3, Table 5, |
|  |  | (*b*) Report category boundaries when continuous variables were categorized | Page 10-14 |  |
|  |  | (*c*) If relevant, consider translating estimates of relative risk into absolute risk for a meaningful time period | Not applicable |  |
| Other analyses | 17 | Report other analyses done—eg analyses of subgroups and interactions, and sensitivity analyses | Page 10-14 | Figure, 3, Figure 5, Figure 6 |
| Discussion | | | |  |
| Key results | 18 | Summarise key results with reference to study objectives | Page 14 | “The principal findings of this study pertain to the potential association of H. pylori with MASLD severity. Our results suggest that H. pylori infection is associated with: (i) cardiometabolic risk factors for MASLD, (ii) steatotic liver phenotype influenced by the prevalence of G-allele PNPLA3, (iii) increased AST and LSM by VCTE in MASLD subjects, (iv) an independent risk of significant/advanced fibrosis by FIB-4 and LSM in MASLD subjects, (v) increased NAS score and fibrosis stage, and (vi) the combination of H. pylori infection and the G-allele PNPLA3 genotype appeared to exacerbate MASLD severity, indicating a synergistic effect.” |
| Limitations | 19 | Discuss limitations of the study, taking into account sources of potential bias or imprecision. Discuss both direction and magnitude of any potential bias | Page 17 | “The limitations of the study include: (1) population studied: Our cohort comprised of South-American population of functional dyspepsia. Though we tried to investigate a cohort whose characteristics could resembled those of the general population, the modality of cohort recruitment did not allow us to affirm that our cohort was fully representative of the general population. However, our cohort was recruited independently from the hypothesis concerning the high risk of MASLD severity” |
| Interpretation | 20 | Give a cautious overall interpretation of results considering objectives, limitations, multiplicity of analyses, results from similar studies, and other relevant evidence | Page 17 | “.Based on the non-invasive nature of the study design in FD-cohort, hepatic steatosis was detected by ultrasonography and fibrosis by FIB-4 score and LSM by VCTE but not liver histology, so the absence of histologic data prevents us from reporting the exact prevalence of steatohepatitis and advanced fibrosis. Noteworthy, LSM by VCTE is the most validated non-invasive method to accurately screen fibrosis in MASLD and can predict the occurrence of liver-related events in MASLD”.  “The retrospective nature of the biopsy-proven MASLD cohort renders it susceptible to recall bias, the duration of H. pylori gastric infection is unknown, and the sample size could be small, but sufficient to show robust statistical significance in major MASLD-histological endpoints.” |
| Generalisability | 21 | Discuss the generalisability (external validity) of the study results | Page 16 | “Our study showed a correlation with H. pylori-infection with elevated non-invasive markers of liver fibrosis by FIB-4 score and LSM by VCTE. Our observation was independent of risk factors of disease severity such as type-II DBT, obesity and G-allele PNPLA3. Noteworthy, this observation was further confirmed in the biopsy-proven MASLD cohort, and appear to have a synergistic effect with G-allele PNPLA3 exacerbating MASLD severity. Confirmation from mechanistic studies is needed to clarify the clinical role of H. pylori infection and fibrogenesis in MASLD” |
| Other Information | | | |  |
| Funding | 22 | Give the source of funding and the role of the funders for the present study and, if applicable, for the original study on which the present article is based | Page 2 | “The study is supported by a grant of the clinical research fund of Fundación HA Barceló. The funders had no role in study design, data collection and analysis, decision to publish, or preparation of the manuscript” |

**Give information separately for cases and controls in case-control studies and, if applicable, for exposed and unexposed groups in cohort and cross-sectional studies.*

**Note:** An Explanation and Elaboration article discusses each checklist item and gives methodological background and published examples of transparent reporting. The STROBE checklist is best used in conjunction with this article (freely available on the Web sites of PLoS Medicine at http://www.plosmedicine.org/, Annals of Internal Medicine at http://www.annals.org/, and Epidemiology at http://www.epidem.com/). Information on the STROBE Initiative is available at www.strobe-statement.org.
